# FedVI: Financial Cross-Domain Federated Learning with Scarce Overlapping Samples via Visual Representation of Heterogeneous Tabular Data and Meta-Optimization

**DOI:** 10.3390/e28060637

**Published:** 2026-06-04

**Authors:** Kaiqing Yuan, Jiang Wu

**Affiliations:** School of Management Science and Engineering, Southwestern University of Finance and Economics, Chengdu 611130, China; 42327124@smail.swufe.edu.cn

**Keywords:** tabular-to-image conversion, federated transfer learning, meta-learning

## Abstract

Federated learning offers a promising approach for cross-institutional financial risk control modeling but encounters two key challenges in practice: feature space heterogeneity and low sample overlap rate. Current federated transfer learning methods often rely heavily on sufficient overlapping samples or explicit feature alignment. However, these approaches frequently result in negative transfer when enforced alignment is applied in highly heterogeneous environments. To address this issue, we propose FedVI, a novel federated transfer learning framework that integrates tabular-to-image conversion and meta-learning mechanisms. Moving beyond conventional methods that rely on sample-level alignment, FedVI employs a federated dual-stream feature alignment strategy to securely reconstruct a unified global feature map across institutions. Subsequently, FedVI integrates federated Image Generator for Tabular Data (IGTD) with tabular Transformer technology to convert one-dimensional tabular data into two-dimensional visual-semantic tensors. These tensors effectively fuse spatial topology and semantic information while embedding an independent Mask channel to explicitly retain the true missingness patterns of features. Finally, FedVI adopts the Model-Agnostic Meta-Learning (MAML) architecture to facilitate global parameter optimization. We evaluated FedVI on the real-world Lending Club credit dataset and Home Credit Default Risk datasets under highly heterogeneous federated settings (i.e., heterogeneous feature spaces across three clients and scarce overlapping samples). The results reveal that FedVI achieves competitive performance against advanced baselines such as FedProx, FedRep, and FedKT, particularly in recall and F1-Score. These findings indicate that FedVI can effectively support cross-domain adaptation under heterogeneous federated learning settings.

## 1. Introduction

Recent advancements in machine learning have achieved breakthroughs in financial risk control, particularly in credit default prediction. However, financial institutions face growing challenges due to the persistent issue of data silos during cross-institutional collaborations. With the implementation of Europe’s General Data Protection Regulation (GDPR) and similar global data privacy laws, direct sharing of raw data or physical merging of datasets between organizations has become increasingly restricted. Federated Learning (FL), a distributed machine learning framework, addresses this problem by enabling participating institutions to retain their original data locally. Nodes within this system train models independently using their own data and exchange only aggregated model parameters or gradients to collaboratively optimize a global model. This approach provides a robust solution for multi-party collaborative modeling in privacy-sensitive sectors like finance [1,2]. Traditional federated learning frameworks demonstrate strong performance in independent and identically distributed (IID) scenarios. However, real-world financial risk control applications face two key challenges: feature space heterogeneity and low sample overlap rate among institutions [3].

Regarding the challenge of feature space heterogeneity, participating institutions differ significantly in their business domains, data acquisition methods, and information system architectures, resulting in locally collected datasets that often exhibit highly heterogeneous feature spaces. This variability in feature spaces substantially hampers the practical implementation of federated learning for multi-party collaborative modeling. Consequently, to enable effective cross-party collaborative modeling and knowledge transfer while safeguarding privacy, integrating heterogeneous client data becomes a critical prerequisite. However, data integration inherently represents a complex and independent research domain within database systems [4]. Central challenges include schema matching—establishing precise correspondences between features across diverse, non-standardized data sources—which remains unresolved due to the absence of unified protocols.

Addressing the challenge of scarce overlapping samples, traditional Vertical Federated Learning (VFL) relies heavily on extensive user overlap across participating institutions to align multi-dimensional features of the same individual. However, in cross-domain settings, distinct institutions typically serve separate customer populations, resulting in scarce overlapping samples. Under such conditions, conventional VFL—which depends on entity matching—becomes infeasible. Conversely, adapting Horizontal Federated Learning (e.g., FedAvg, FedProx [5]) by restricting training to minimal shared features and sparse overlapping samples introduces critical drawbacks: it risks losing valuable local private data, impedes the generation of high-quality shared representations, and ultimately causes significant performance decline.

Existing Federated Transfer Learning (FTL) approaches aim to address feature space heterogeneity and low sample overlap rate but face two key limitations. First, most models are restricted to bilateral collaboration [6], limiting their scalability to multiple clients. Second, traditional methods typically rely on the assumption of relatively high sample overlap [7]. However, as the number of participating institutions grows, the proportion of jointly owned samples diminishes significantly, severely hindering the practical applicability of current techniques. Consequently, developing a multi-party federated framework capable of accommodating cross-domain adaptation while reducing reliance on strict sample alignment emerges as the central objective of this study.

To address the dual dependency of traditional federated transfer learning (FTL) on high-overlap shared features and overlapping samples, to the best of our knowledge, this paper introduces the first federated transfer learning framework, FedVI, which integrates tabular-to-image conversion and meta-learning mechanisms. The proposed FedVI framework departs from traditional sample-level alignment approaches, reframing the challenge of cross-domain tabular joint modeling as a cross-modal visual pattern recognition problem. Specifically, FedVI adopts a semantic-and-statistical dual-stream alignment strategy to securely reconstruct the global feature map. Subsequently, it integrates Federated IGTD (a tabular data image generator) and tabular Transformer technologies to transform abstract one-dimensional tabular data into two-dimensional representations that combine feature distributions and local interaction patterns. Finally, by leveraging a model-agnostic meta-learning framework, the model achieves enhanced rapid adaptation to cross-domain tasks, enabling effective knowledge sharing and transfer.

In summary, FedVI offers a feasible and effective solution to mitigate negative transfer challenges in federated transfer learning for cross-domain heterogeneous tabular data scenarios, showcasing significant practical potential and theoretical value. The main contributions of this paper are summarized as follows:

1. For cross-domain collaboration in financial institutions using federated learning, to the best of our knowledge, we propose the first federated transfer framework that integrates tabular-to-image conversion and meta-learning mechanisms. This approach addresses two key challenges: feature space heterogeneity across domains and low sample overlap rate between datasets.
2. To address feature space heterogeneity, we propose a visual feature alignment mechanism that integrates dual-stream feature alignment strategies with federated IGTD and tabular Transformers. This approach transforms 1D heterogeneous tabular data into 2D visual-semantic tensors. Consequently, it enables precise feature alignment in the visual space without compromising data privacy.
3. To address scenarios with low sample overlap rate, we propose a federated meta-learning strategy designed for scarce overlapping samples. Unlike conventional approaches that rely on sample alignment and standard federated aggregation, our method employs federated meta-learning (FML) aggregation to facilitate efficient cross-domain knowledge sharing and enable personalized model adaptation, even when overlapping samples are scarce.

## 2. Related Work

Recently, with the growing awareness of data privacy protection and the increasing prominence of data silo issues, federated learning has emerged as a key research direction for multi-party collaborative modeling. As a distributed machine learning paradigm that enables joint training without centralized raw data, federated learning has demonstrated broad application potential across finance, healthcare, government, and other domains [8,9].

### 2.1. Federated Learning Under Feature Space Heterogeneity

Traditional federated learning methods, such as FedAvg [1], FedProx [5], and FedBN [10], primarily focus on homogeneous data environments, assuming all clients share identical feature spaces. This assumption limits their effectiveness in cross-domain scenarios where feature structures diverge. In practical cross-organizational collaborations, disparities in data sources, business processes, and storage formats frequently lead to feature space heterogeneity—clients may possess distinct feature sets or variable field names. These discrepancies prevent direct parameter sharing, significantly impairing the efficacy of federated modeling. In federated learning research, studies conventionally define moderate heterogeneity as environments with feature overlap rates ranging from 30% to 50%, while categorizing rates below 30% as high heterogeneity. This paper extends this framework by addressing extreme heterogeneity, rigorously defining it as settings where the feature overlap (i.e., the intersection size of feature spaces normalized by the total features across all parties) does not exceed 20%. To mitigate this challenge, prior studies have explored cross-feature transfer techniques, including feature mapping, subspace alignment, and shared encoder approaches. For example, HeteroFL [11] enables participants to adopt distinct model architectures and collaborates through knowledge distillation. FedMA [12] aligns neural network layers during aggregation using model matching algorithms. HyFDCA [13] leverages Fenchel duality and homomorphic encryption to ensure privacy-preserving collaborative training. FedMD [14] facilitates knowledge sharing between differently structured models using public datasets via knowledge distillation. Federated Mutual Learning [15] supports cross-feature knowledge transfer through mutual supervision among models with varied feature spaces during training. While these approaches address certain aspects of heterogeneity, they primarily rely on assumptions of partial feature overlap or semantically aligned domains. Consequently, their effectiveness diminishes in highly heterogeneous real-world scenarios where such conditions are absent. To address significant heterogeneity challenges, the proposed FedVI employs a privacy-preserving dual-stream feature alignment strategy, which reconstructs a global feature map without sharing any underlying raw features or data. This approach eliminates reliance on feature overlap, alleviates data silo issues arising from extreme heterogeneity, and facilitates effective cross-domain knowledge transfer and collaborative modeling in highly diverse environments.

### 2.2. The Challenge of Federated Modeling Under Scenarios with Low Sample Overlap Rate

A significant challenge emerges in scenarios involving low sample overlap rate, particularly within vertical federated learning (VFL). Traditional VFL methods depend heavily on sample overlaps across institutions—for instance, cases where the same user has records in both banking and insurance systems. These overlaps enable Private Set Intersection (PSI) and other alignment techniques to facilitate feature concatenation and joint modeling [16]. Classical research in federated learning typically assumes that participating parties share over 50% of their samples to ensure alignment effects. Existing studies addressing “low overlap rates” often set this threshold at 30%. In contrast, this study examines increasingly challenging cross-domain scenarios by quantitatively defining them as those with ≤20% shared sample proportions. However, in cross-domain applications characterized by distinct customer groups with minimal or no overlapping samples, training approaches reliant on sample alignment become impractical. To address this issue, researchers have proposed solutions such as encrypted matching, client selection, and pseudo-identifier mechanisms to improve sample-level alignment [17,18,19]. While these approaches aim to mitigate the limitations of traditional methods, they introduce additional communication overhead and potential privacy risks. Furthermore, their effectiveness remains limited under conditions of extremely low sample overlap rate. To address the challenge of low sample overlap rate, the proposed method abandons conventional sample-level alignment techniques. Drawing inspiration from Zhu et al.’s tabular data image generator (Image Generator for Tabular Data, IGTD) [20], this study innovatively adapts the IGTD framework to federated collaborative settings. Unlike the original approach, which relies on centralized data, our work reconstructs global feature correlations through secure aggregation of local statistics across institutions. By eliminating the need for physical or encrypted sample matching, IGTD projects independent samples from diverse institutions onto a shared global 2D visual space. This facilitates representation-level knowledge fusion via personalized fine-tuning strategies, enabling effective collaboration without compromising data privacy. By reducing dependency on overlapping samples, IGTD addresses key challenges in federated learning—mitigating overfitting in localized models under highly heterogeneous conditions and substantially improving the generalization performance of collaborative global models.

### 2.3. Federated Transfer Learning and Meta-Learning

To address both feature space heterogeneity and low sample overlap rate, recent research has proposed integrating transfer learning with federated learning, termed federated transfer learning (FTL). This approach typically leverages pre-trained models, auxiliary models, self-supervised learning mechanisms, or representation alignment strategies to enhance cross-party knowledge transfer in heterogeneous environments. For instance, Nguyen Tan et al. [21] enhanced model performance by integrating convolutional neural networks pre-trained on ImageNet with locally applied SMOTE oversampling techniques. FedProc [22] adopts a prototype learning framework wherein clients’ local class prototypes act as shared intermediates. Through global prototype alignment and contrastive learning, this approach enables semantic representation transfer across clients, improving generalization in non-IID settings. MOON [23] proposes local contrastive learning objectives to reinforce global consistency. The pFedKT framework [24] introduces a bidirectional knowledge transfer mechanism through mutual knowledge distillation between global servers and local clients, enhancing model perception and adaptability to localized data distributions. A distinct research direction integrates meta-learning approaches. For example, Model-Agnostic Meta-Learning (MAML) [25], a foundational algorithm, achieves strong transfer performance across multi-task scenarios. It has been adapted to federated systems to enable rapid adaptation to data distribution shifts [26].

Current federated transfer, personalization, and meta-learning approaches predominantly succeed under conditions of small-scale data or limited heterogeneity. These methods remain reliant on homogeneous feature space assumptions or depend on third-party public datasets to mediate knowledge distillation, which hinders their effectiveness in scenarios involving low sample overlap rate and feature space heterogeneity simultaneously. The proposed FedVI framework addresses these limitations by integrating tabular-to-image conversion and meta-learning mechanisms, thereby establishing a novel cross-domain personalization paradigm for heterogeneous tabular data. By reducing dependence on high-overlap features/samples—traditional requirements of federated transfer learning—the FedVI framework provides a robust adaptive framework for cross-domain federated modeling. Further technical details of this implementation are described in Section 3.

## 3. Proposed Method

### 3.1. Problem Formulation

In the cross-domain federated learning framework for financial credit default prediction tasks, the federated learning system comprises N clients, each client $C_{i}$ possessing an independent local dataset.

(1) $$D_{i}=\{\left(x_{i}^{k},y_{i}^{k}\right){\}}_{k=1}^{n_{i}},\;\;x_{i}^{k}\in R^{d_{i}}{,\;\;y}_{i}^{k}\in \left\{0,1\right\}\;\;\;$$

where $d_{i}\neq d_{j}$ denotes that the feature dimensions vary across different clients. Let $ID(D_{i})$ denote the sample space of client $C_{i}$. In highly heterogeneous cross-domain scenarios, clients satisfy a low sample overlap rate θ constraint (set to 20% in this study), i.e., for any i≠j:

(2) $$|ID(D_{i})\cap ID(D_{j})|\leq \theta$$

Participants encounter two primary challenges arising from variations in business contexts and data collection methodologies: 1. Feature space heterogeneity: disparities in data collection lead to inconsistent feature dimensions and semantic nuances across client datasets. This variability prevents effective training through conventional parameter sharing approaches. 2. Low sample overlap rate: collaborating institutions typically serve distinct customer populations, resulting in scarce overlapping samples across datasets. Traditional methods relying on database keys for alignment or cross-table joining become impractical under these conditions. As the scale of client participation grows, sample overlap diminishes further, leaving most practical applications without sufficient commonalities for reliable cross-domain analysis. Consequently, conventional transfer learning techniques dependent on sample alignment or joint supervision prove ineffective in establishing meaningful inter-domain relationships.

To address these dual challenges, this paper proposes a novel framework. For feature space heterogeneity, we design a dual-stream feature alignment strategy and a tabular-to-image conversion to reconstruct global features across statistical and semantic dimensions, mapping them into dimensionally aligned multi-channel 2D visual representations for unified modeling in visual spaces. To tackle low sample overlap rate, we move beyond traditional federated aggregation strategies and innovatively propose federated meta-learning (FML), which integrates global meta-model aggregation with local personalized fine-tuning mechanisms. This approach enables efficient collaborative modeling and cross-domain knowledge transfer, even under extreme conditions with scarce overlapping samples.

### 3.2. The Overall Framework

**Figure 1:**
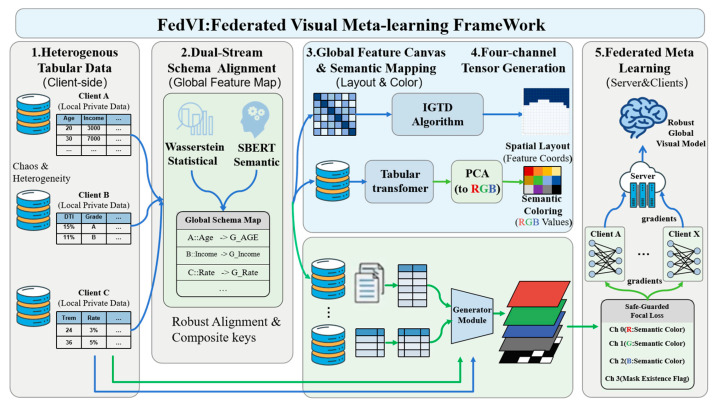
Architecture of FedVI.

The proposed FedVI framework addresses challenges in joint training across multiple heterogeneous tabular datasets. As illustrated in Figure 1, the framework comprises four core phases. Phase 1: Federated Schema Alignment: To resolve the “data silo” issue stemming from discrepancies in feature naming and dimensionality across participating institutions, this phase employs a dual-stream alignment strategy grounded in semantic and statistical distribution matching. By avoiding direct exchange of raw underlying samples, it securely reconstructs and unifies the global heterogeneous feature space. Phase 2: Global Feature Canvas Construction and Semantic Coloring: To address scenarios with scarce overlapping samples, the federated IGTD algorithm is employed to reconstruct the 2D spatial coordinates of global features. Concurrently, tabular Transformer training is utilized to extract deep semantic representations for RGB channels. This dual approach enables the mapping of abstract one-dimensional tabular features into visual layouts that integrate both spatial topology and semantic information. Phase 3: The construction of a four-channel tensor incorporating presence Mask: This phase tackles noise introduced by imputed missing values (e.g., zero-imputation or mean substitution) in tabular data. An innovative solution involves introducing an independent Mask channel that explicitly preserves the original missing-data patterns, thereby mitigating distortion caused by conventional imputation methods. By integrating these components with the RGB channels, a 4-channel tensor is constructed, enabling the model to perceive the actual data presence status. Phase 4: Federated Visual Training: To address overfitting caused by significant data heterogeneity, a Reptile-based federated meta-learning algorithm is employed to aggregate global knowledge. This approach, combined with a compact convolutional neural network (CNN) optimized for small canvas sizes, allows for personalized fine-tuning at the client side, ultimately facilitating efficient and robust collaborative predictions.

The FedVI framework is a closed-loop system designed for extreme heterogeneous environments, where all three core components are essential. While the federated IGTD algorithm constructs the spatial topology of global features without requiring sample-level alignment, it produces only single-channel scalar values, thereby neglecting intricate implicit semantic relationships between financial features. To address this limitation, a tabular Transformer is introduced to “semanticize” the data by converting one-dimensional tabular features into two-dimensional visual semantic tensors. This transformation allows subsequent convolutional models to simultaneously capture numerical intensity and underlying semantic patterns. However, even after constructing these 2D visual semantic tensors, local client data remains highly fragmented under extremely limited sample overlap. Conventional federated aggregation methods would exacerbate distribution shifts, resulting in parameter divergence and negative transfer. The framework employs federated meta-learning as an optimization mechanism. By leveraging reconstructed 2D visual semantic tensors, meta-learning identifies globally generalized initialization parameters that are applicable across heterogeneous clients. Specifically, federated IGTD redefines the spatial topology of heterogeneous features, the tabular Transformer integrates deep semantic information, and meta-learning enables efficient cross-domain knowledge transfer under heterogeneous data distributions.

### 3.3. Federated Dual-Stream Schema Alignment

To address feature space heterogeneity, we designed a dual-stream alignment mechanism combining semantics and statistics. Financial heterogeneous field alignment cannot be reliably achieved through single-source similarity methods alone. Sole reliance on semantic similarity risks challenges such as synonyms with distinct institutional naming conventions or homonyms with divergent meanings across organizations. Conversely, prioritizing statistical similarity may erroneously align fields with analogous data distributions but disparate business implications. To address these limitations, this study proposes a joint modeling framework that integrates semantic and statistical analysis. Specifically, the semantic component extracts field names and their contextual business definitions, while the statistical component analyzes feature value distributions and structural patterns. By concurrently incorporating both modalities, the proposed approach enforces mutual constraints during schema alignment, enhancing alignment accuracy across heterogeneous domains. Specifically, the semantic stream leverages SBERT to extract semantic vector embeddings $e_{sem}$ for column names and computes cosine similarity. For privacy-preserving feature alignment in credit client data, the statistical stream allows each client to upload only statistical vectors $e_{stat}$ based on quantiles, zero-value ratios, negative-value ratios, unique-value ratios, and skewness. The server then calculates a composite similarity matrix S.

(3) $$S_{ij}=\alpha \cdot {Sim}_{sem}(e_{sem}^{\left(i\right)},e_{sem}^{\left(j\right)})+(1-\alpha )\cdot {Sim}_{stat}(e_{stat}^{\left(i\right)},e_{stat}^{\left(j\right)})$$

As shown in Equation (3), ${Sim}_{stat}$ represents comprehensive statistical similarity. To accurately measure differences across various statistical features, this paper employs the Wasserstein distance for measuring continuous distribution shape vectors composed of quantiles, while for structured feature vectors consisting of scalar metrics such as zero-value rate, negative-value rate, unique-value ratio, skewness, etc., Euclidean distance is used for calculation. ${Sim}_{sem}$ denotes semantic similarity calculated via cosine similarity. α represents the fusion weight assigned to the integration of semantic and statistical similarities. In this implementation, α is configured to 0.6, establishing contribution ratios of 0.6 and 0.4 for semantic and statistical similarities, respectively. This configuration leverages the inherently rich business semantics of financial domain names, enabling semantic similarity to dominate the alignment process. Statistical similarity is retained as a supplementary validation mechanism at the distribution level, mitigating risks of misalignment that could arise from reliance on field names alone. Notably, α functions as an empirically derived hyperparameter and remains constant across subsequent experimental evaluations. The final global feature mapping table M is generated through hierarchical clustering, resolving structural and semantic barriers.

**Figure 2:**
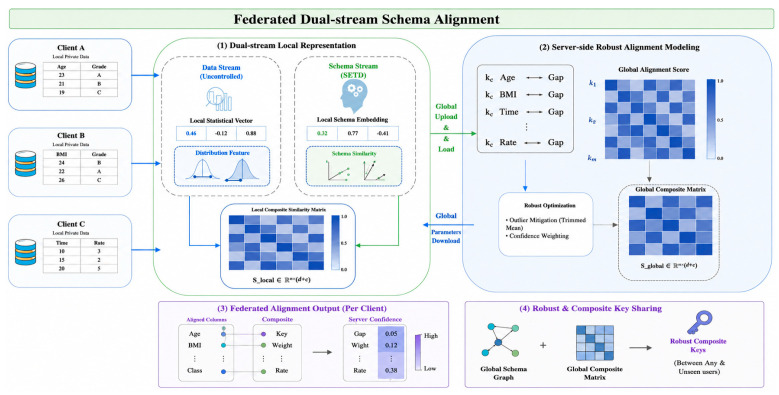
Dual-Stream Schema Alignment for Heterogeneous Financial Features.

As illustrated in Figure 2, the Dual-Stream Schema Alignment Module comprises two distinct streams: the semantic stream and the statistical stream. The semantic stream identifies similarities in field names and business semantics, whereas the statistical stream captures feature distribution-level similarities. Together, these components generate a global feature mapping table, which serves as the foundational basis for constructing a unified two-dimensional canvas for subsequent federated IGTD.

### 3.4. Global Feature Canvas Construction and Semantic Coloring

#### 3.4.1. Federated IGTD via Gram Matrix Aggregation

To exploit the inherent feature extraction capabilities of convolutional neural networks (CNNs) for capturing local interaction patterns in credit-structured data, FedVI employs the IGTD algorithm to map one-dimensional feature vectors xi into two-dimensional images of size H × W. The IGTD method minimizes the discrepancy between the feature ranking matrix R and the pixel ranking matrix Q by identifying an optimal allocation scheme that assigns features to pixel positions such that similar features are spatially proximate in the image. Matrix R records ascending rankings of feature similarities based on Pearson correlation coefficients, while matrix Q encodes Euclidean distance rankings between pixel coordinates. The optimization objective function is formulated as shown in Equation (4):

(4) $$err(R,Q)=\sum_{i=2}^{N}\sum_{j=1}^{i-1}diff(r_{i,j},q_{\pi (i),\pi (j)})$$

Equation (4) represents the feature-pixel sorting matching objective from the original IGTD framework. The federated learning approach introduced in this paper maintains the core optimization structure of IGTD’s sorting mechanism while addressing the challenge of inaccessible globally correlated feature matrices inherent to federated settings. where N represents the total number of global features, where diff(⋅,⋅) denotes the absolute difference used to quantify the deviation within the lower triangular matrix rank sequence. The algorithm iteratively swaps pixel allocation positions of features to minimize errors. Under federated transfer learning scenarios, participating financial institutions only possess heterogeneous subsets of local features, making it impossible to construct a global feature ranking matrix containing all client features locally. Due to strict privacy protection requirements in federated learning, uploading raw data to build the global feature ranking matrix R is prohibited. To create a global common two-dimensional image incorporating all client features while preserving customer privacy using IGTD, we design a correlation reconstruction mechanism based on Gram matrix aggregation.

To prevent data leakage, each client k exclusively uses its local training dataset to compute correlation statistics during collaborative processes. Under non-IID federated settings, this approach generates an approximate estimate of the global correlation structure instead of achieving a precise unbiased reconstruction of the centralized correlation matrix. Missing values are addressed via mean imputation rather than zero-imputation, thereby preventing the artificial introduction of excessive zero values that could distort the authentic covariance relationships between features. Furthermore, to prevent positive and negative correlations from canceling each other out due to inconsistent categorical encoding, each client deterministically standardizes categorical feature encoding using Deterministic Hash Encoding.

After preprocessing, each client k only locally computes and uploads two statistics: the feature sum vector $S_{k}=\sum X_{k}$ and the Gram matrix $G_{k}=X_{k}^{T}X_{k}$, since $G_{k}$ is the Gram matrix representing the inner products of the features without specific sample information. Considering the non-overlapping nature of feature spaces in heterogeneous scenarios, the joint statistics of features i and j cannot be simply divided by the total global sample size $N_{total}$. To address this, the server introduces a feature availability mask vector $m^{\left(k\right)}\in \{0,1{\}}^{N}$ (where 1 indicates the presence of a feature at client k and 0 otherwise). Let client k have $N_{k}$ samples; the server first aggregates the masks to reconstruct the feature-specific effective sample size vector c and the pairwise valid sample count matrix C.

(5) $$c=\sum_{k}N_{k}m^{\left(k\right)},\;C=\sum_{k}N_{k}(m^{\left(k\right)}(m^{\left(k\right)}{)}^{T})$$

Based on the above effective sample size, the server can aggregate local statistics from various clients to compute the expectation vector E[X] of global features and the product expectation matrix $E[X^{T}X]$.

(6) $$E[X]=(\sum_{k}S_{k})⊘c$$

(7) $$E[X^{T}X]=(\sum_{k}G_{k})⊘C$$

where ⊘ denotes element-wise division (Hadamard Division) of matrices or vectors. Finally, based on the definition of covariance, the federated paired covariance matrix estimated on the server-side $Cov_{global}$ can be derived as:

(8) $$Cov_{global}=E[X^{T}X]-E[X]E[X{]}^{T}$$

Furthermore, by dividing the covariance matrix ${Cov}_{global}$ by the outer product of each feature’s standard deviations, the global Pearson correlation coefficient matrix $Corr_{global}$ is derived as input for the IGTD algorithm. The method does not assert strict equivalence to centralized computation under non-IID (non-independent and identically distributed) conditions. Instead, it approximates the global feature correlation structure without exchanging raw data samples. This approximation enables the construction of a feature distance matrix, which guides the Image Generator for Tabular Data (IGTD) algorithm to generate a unified two-dimensional feature visualization. By minimizing the error between the feature ranking matrix R and pixel spatial distance Q, the optimal pixel coordinate mapping π:F → (h, w)∣1 ≤ h ≤ H, 1 ≤ w ≤ W is obtained. In federated heterogeneous settings, discrepancies in client data distribution and the presence of features that co-occur exclusively on specific clients can cause the covariance matrix aggregated by the server to diverge from the true covariance under ideal centralized conditions. Consequently, this paper posits the matrix derived via Equation (8) as a federated covariance estimate, distinct from the precise global covariance. This estimate primarily aims to furnish prior structural insights into feature correlations for IGTD, rather than reconstructing comprehensive centralized statistical data. To address potential estimation biases, three mitigation strategies are integrated into the implementation: first, correlations are computed exclusively using training data to prevent test information leakage; second, categorical variables are encoded deterministically via hash functions to mitigate correlation discrepancies arising from inconsistent client-side encoding practices; third, mean imputation is applied during correlation calculations to avoid introducing artificial aggregation points through zero-imputation. While these measures cannot entirely eliminate non-IID bias, they enhance the stability of federated covariance matrix estimation. Notably, this framework satisfies strict data privacy constraints: during the IGTD layout construction phase, clients upload only Gram matrices $G_{k}\in R^{N\times N}$ and sum of feature values $S_{k}$. Since the Gram matrix $G_{k}$ aggregates inner products in the feature dimension and the number of features is far smaller than the sample count $N\ll n_{k}$, attackers cannot reconstruct the original sample matrix $X_{k}$ from $G_{k}$. Additionally, categorical features undergo Deterministic Hash Encoding, further obscuring raw semantic information and ensuring compliance with the “data localization” principle.

In IGTD, the allocation of feature-pixel positions constitutes a combinatorial optimization problem, formally akin to quadratic assignment problems. As the number of global features grows, the computational cost of exhaustively seeking the optimal layout escalates exponentially. To address this challenge, this paper employs an iterative swap-based heuristic strategy to generate approximate solutions. The algorithm proceeds by iteratively attempting pairwise exchanges of pixel positions: if a swap reduces the discrepancy between the feature ranking matrix and the pixel distance ranking matrix, it is accepted; otherwise, the current configuration is retained. The process concludes when either the maximum iteration limit is attained or consecutive steps yield diminishing returns (i.e., error reduction falls below a predefined threshold). While this method does not ensure global optimality, it consistently produces reproducible 2D layouts under practical computational constraints.

**Figure 3:**
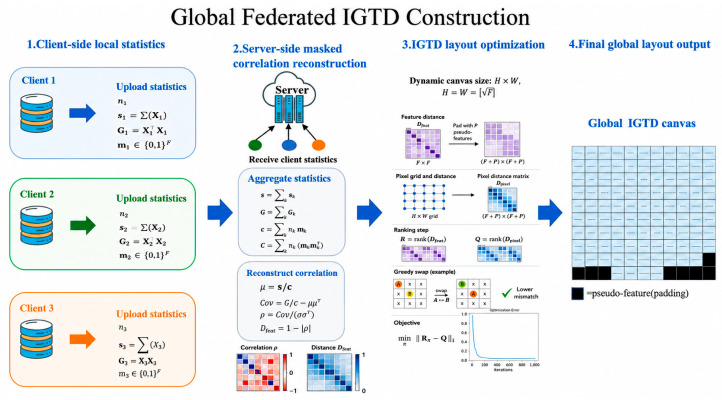
Federated IGTD-Based Global Feature Canvas Construction.

As illustrated in Figure 3, the federated IGTD module constructs a global two-dimensional feature canvas without requiring clients to share raw data. Specifically, each client uploads only local statistics, including feature-sum vectors, Gram matrices, and feature-availability masks, to the server. Using this aggregated information, the server then reconstructs global feature correlations. Finally, the IGTD algorithm arranges highly correlated features adjacently, facilitating efficient local feature interaction learning by convolutional neural networks (CNNs).

For example, in the financial credit scenario, if the features “annual income” and “employment duration” have a high correlation in the original statistical distribution, their distance rank $r_{i,j}$ in the feature ranking matrix R will be smaller. The IGTD algorithm will perform multiple iterations to assign these two features to adjacent pixels on the canvas, enabling the CNN convolution kernel to capture the hidden nonlinear correlation between them.

#### 3.4.2. Feature Semantic Coloring via Tabular Transformer

After determining the spatial topological position of features, this study employs a tabular Transformer to perform semantic coloring on the features, overcoming the limitation of pure numerical features lacking deep semantic associations. The network utilizes an input processor to perform contextual joint encoding of column names and cell values. Specifically, for categorical features $x_{c}$, the model concatenates the column name with the specific category description into a natural language sequence, which is directly mapped via a tokenizer to a semantic embedding $E_{c}$. For continuous numerical features $x_{u}$, the network adopts a column embedding multiplication strategy to align them with the semantic space.

(9) $$E_{u}=x_{u}\times E_{u,col}\;$$

where $E_{u,col}$ represents the initial semantic embedding of the corresponding column name for feature $x_{u}$. After encoding all categorical and continuous features, these feature embeddings are aligned to 128 dimensions through layer normalization and linear projection, then concatenated into an initial feature sequence representation $Z^{0}$. To capture complex interactions among heterogeneous financial features, $Z^{0}$ is fed into a multi-head self-attention mechanism for global encoding, producing the attention representation $Z_{att}^{l}$. To further filter redundant feature interactions and focus on core risk control signals, the model introduces a gating layer $g^{l}=\sigma (Z_{att}^{l}w^{g})$ after the attention layer, which reassigns feature attention weights through the following mechanism:

(10) $$Z^{l+1}=Linear((g^{l}⊙Z_{att}^{l})⊕Linear(Z_{att}^{l}))$$

During the model training phase, each client employs vertically partitioned contrastive learning to train the tabular Transformer. By maximizing semantic expression consistency of the same financial entity across different feature views, the model is encouraged to deeply capture latent financial relationships between variables. After multiple federated communication rounds and local parameter aggregation, the server holds an optimal tabular Transformer model. Following the completion of IGTD’s canvas coordinate layout, we input every feature name from the global feature map into the aggregated Transformer encoder to extract their semantic embedding vectors CLS($\in R^{128}$) at the server side. This paper employs Principal Component Analysis (PCA) to orthogonally project the 128-dimensional semantic embeddings into a three-dimensional feature subspace. Subsequently, Min-Max normalization is applied to precisely align these reduced features with the baseline RGB color channel values of the pixel grid. This novel semantic coloring mechanism offers two key advantages: it provides subsequent CNN models with a highly discriminative multi-channel visual receptive field, and it fundamentally addresses the limitations of conventional tabular-to-image conversion methods, which rely on single-channel grayscale representations.

**Figure 4:**
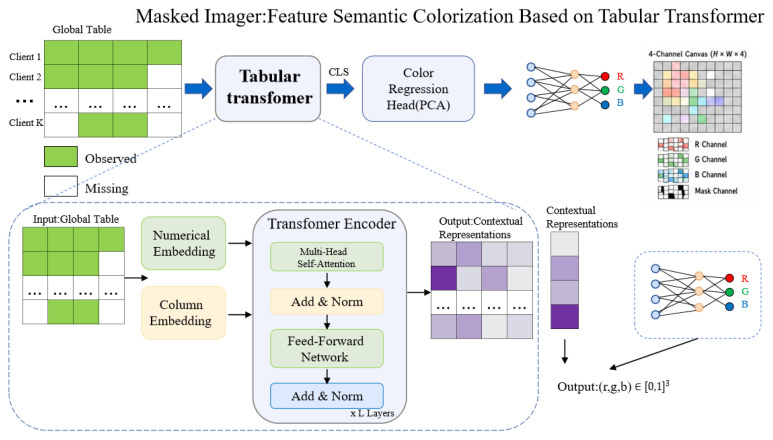
Semantic Coloring of Global Feature Canvas via Tabular Transformer.

As illustrated in Figure 4, the Transformer semantic coloring module maps RGB semantic colors to each feature on a 2D canvas. While federated IGTD primarily encodes statistical topological relationships between features, the Transformer additionally extracts deeper semantic information from field names and their corresponding values. This integrated approach ensures that the resulting visual representation combines spatial organization with distinct semantic categorization.

**Figure 5:**
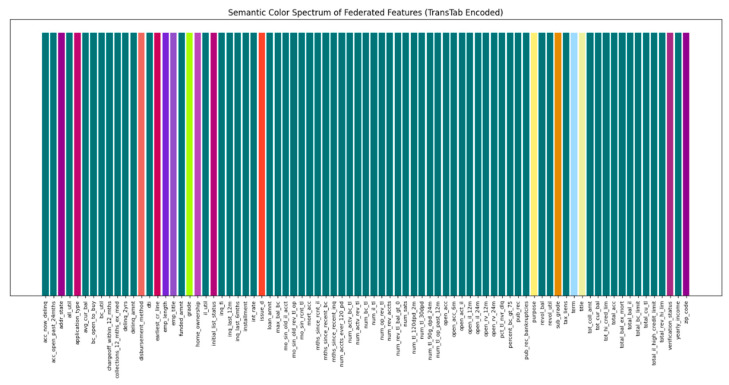
Visualization of the semantic color spectrum generated by the tabular Transformer.

In addition, the semantic color spectrum generated from the Transformer-derived feature embeddings is visualized in Figure 5, which illustrates the RGB semantic assignments across global features.

### 3.5. The Construction of a 4-Channel Tensor with an Integrated Presence Mask

After completing the spatial topological arrangement and semantic color allocation of global features, the FedVI framework converts each client’s local heterogeneous tabular data samples into a tensor of dimension 4×H×W. This tensor is constructed by orthogonally concatenating the semantic numerical channels (RGB) with the presence Mask channel. Specifically, the first three channels encode numerical intensity and semantic attributes. For a given feature in a sample, its RGB pixel values are dynamically computed by taking the scalar product of the normalized feature value $x_{norm}$ and the baseline color vector $Color_{semantic}$. This color vector is derived by applying dimensionality reduction to the high-dimensional semantic features extracted via the tabular Transformer. The Mask channel explicitly indicates feature presence. If the feature exists in the sample (even if its value is 0), the fourth Mask channel is set to 1; otherwise, it is set to 0 for missing features (NaN). This design allows convolutional neural networks to leverage the Mask channel to capture sparse patterns in the data, preventing distribution shifts caused by zero-imputation. For example, consider the feature “historical default record,” which has been assigned to coordinate (10, 12) via IGTD and mapped to red as its semantic color. For a sample with a normalized value of 0.9 indicating a default, the pixel would be generated as [0.9, 0, 0, 1]. If the client’s sample lacks this feature entirely, the Mask channel is 0, resulting in [0, 0, 0, 0]. Although mean imputation is utilized during the IGTD coordinate generation to preserve feature covariances, the final visual tensor fed into the CNN explicitly employs this zero-imputation RGB representation, complemented by the binary Mask channel. This joint representation of semantics and existence guides the CNN model to automatically identify and handle federated heterogeneity-induced data missingness during convolution.

**Figure 6:**
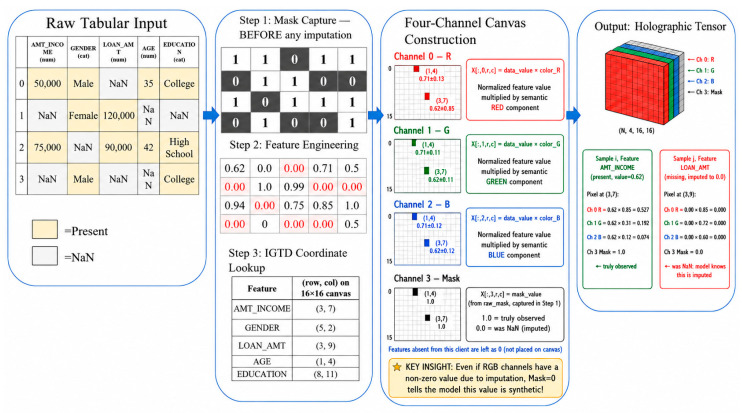
4-Channel Visual Semantic Tensor Generation with Presence Mask.

As illustrated in Figure 6, the four-channel tensor comprises RGB semantic value channels and a Mask channel. The first three channels encode both feature intensity values and semantic attributes, whereas the fourth channel specifies whether a feature is present in the current client. By differentiating between genuine zero values and missing features, the Mask channel mitigates misleading effects arising from zero-imputation techniques.

### 3.6. The Federated Meta-Learning Optimization Process

After generating the four-channel tensor, the model enters the federated training phase. Within the FedVI framework, each financial institution operates with distinct data distributions and heterogeneous feature spaces. Consequently, local training for individual clients can be conceptualized as independent tasks. Federated meta-learning aims to learn a generalized initialization parameter θ across these tasks, enabling rapid adaptation to new clients’ data distributions via minimal gradient updates. Formally, the federated meta-learning objective of FedVI is defined as:

(11) $$\underset{\theta }{\min}\sum_{i=1}^{N}p_{i}L_{i}\left(U_{i}^{K}(\theta )\right)$$

where:

(12) $$p_{i}=\frac{n_{i}}{\sum_{j=1}^{N}n_{j}}$$

(13) $$U_{i}^{K}(\theta )=\theta_{i}^{t,K}$$

where $p_{i}$ denotes the weight of client i, $L_{i}$ denotes the local loss function on client i, $n_{i}$ is its number of local samples, and $U_{i}^{K}(\theta )$ represents the parameters obtained by client i after K local update steps, initialized with the global parameters θ. This objective emphasizes post-update performance rather than the average performance of the initial parameters across all clients. Consequently, this formulation is better suited for the financial scenarios addressed by FedVI, which are characterized by low sample overlap rates and heterogeneous feature spaces.

At the beginning of each communication round t, the server distributes the current global parameters $\theta_{global}^{t}$ to all clients k as the initial weights for their local models, i.e., $\theta_{k,0}^{t}=\theta^{t}$. Given the severe class imbalance in scenarios like financial risk control, traditional cross-entropy loss functions tend to bias models toward the majority class. To address this issue, we replace the standard cross-entropy loss with Focal Loss as the optimization objective $L_{k}$ during local training.

(14) $$L_{k}(p_{t})=-\alpha (1-p_{t}{)}^{\gamma }\log(p_{t})$$

In Equation (14), $p_{t}$ represents the predicted probability of the model for the ground-truth class. α and γ are hyperparameters that can be set. The federated server maintains a shared global model parameter θ, which serves as the initialization for all client models. At the start of each communication round, the server distributes the current θ to all participating clients. During the local update phase e=1,…,E, clients perform parameter updates using the Adam optimizer:

(15) $$\theta_{k,e}^{t}=\theta_{k,e-1}^{t}-\eta_{inner}\nabla L_{k}(\theta_{k,e-1}^{t})$$

where $\eta_{inner}$ represents the local learning rate, and the gradients are optimized utilizing the Adam optimizer during implementation. This robust training based on Focal Loss ensures that models across clients can still extract effective feature representations under heterogeneous and imbalanced data distributions. After E local update steps, each client k uploads the updated model parameters $\theta_{k,E}^{t}$ to the central server. The central server then aggregates all received parameters using the FedReptile algorithm.

(16) $$\theta^{t+1}=\theta^{t}+\eta_{meta}\cdot \left(\frac{1}{K}\sum_{k=1}^{K}(\theta_{k,E}^{t}-\theta^{t})\right)$$

where K represents the total number of clients participating in this round of communication, $\eta_{meta}$ is the meta-learning rate that gradually decays with training rounds. This update rule seeks an optimal initialization parameter that facilitates rapid local fine-tuning across all clients, rather than a simple parameter average. The federated meta-learning optimization process ensures that only model gradients or parameters are transmitted between clients, while raw tabular data and its mappings remain strictly localized, thereby preserving data privacy and enhancing model generalization under heterogeneous data distributions.

**Figure 7:**
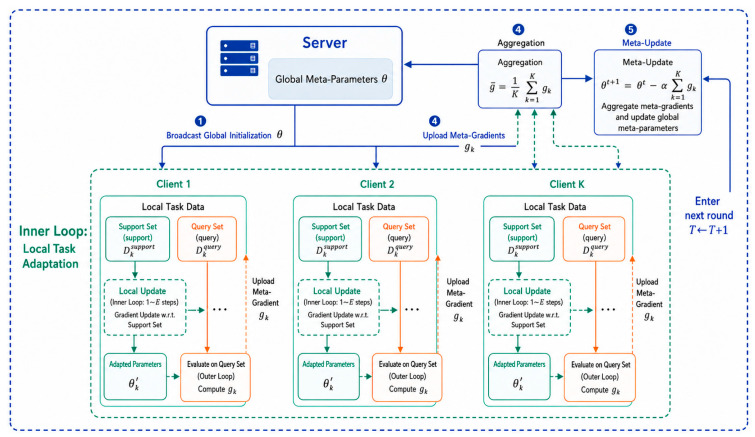
Federated Meta-Learning Optimization with Reptile-Based Aggregation.

As shown in Figure 7, the federated meta-learning module consists of three steps: global initialization, local rapid adaptation, and server-side meta-aggregation. FedVI treats each client as an independent yet related learning task, leveraging Reptile-style updates to learn a globally initialized parameter set that adapts easily across different clients. This design helps mitigate negative transfer issues caused by low sample overlap rates and feature space heterogeneity.

The implementation of the FedVI framework and the corresponding experimental scripts were developed using PyCharm 2024.3.3 as the integrated development environment.

## 4. Experiment

### 4.1. Dataset Description and Data Preprocessing

The dataset used in this experiment is the real-world credit dataset provided by Lending Club. The dataset encompasses a large number of individual loan applications and their subsequent repayment histories, widely utilized in credit assessment and financial risk modeling. We first preprocessed the original dataset by: (1) removing columns with missing values exceeding 30%; (2) eliminating features devoid of practical significance for modeling (e.g., user IDs, descriptive text); (3) removing all post-disbursement features that might leak repayment information; (4) normalizing remaining numerical features and applying categorical encoding to categorical variables. Additionally, to further validate FedVI’s generalization capability across different financial credit data distributions, this paper introduces the Home Credit Default Risk dataset as an auxiliary experimental dataset. This dataset originates from real consumer credit scenarios, with the task objective being to predict default risk based on applicants’ application information, credit history, and repayment-related features. Similar to the Lending Club dataset, the Home Credit dataset exhibits high-dimensional heterogeneous tabular features, a large number of categorical variables, significant missing value proportions, and class imbalance characteristics, making it suitable for supplementary validation in cross-domain financial risk modeling. This study adopts the same data preprocessing pipeline as applied to the Lending Club dataset. Subsequently, multi-client data is constructed following identical federated heterogeneous partitioning strategies to verify FedVI’s robustness under varying overlap conditions.

The main feature categories and their descriptions used in the heterogeneous client setting are summarized in Table 1.

### 4.2. Dataset Segmentation

In the experiment, we use the Lending Club dataset as an example, dividing it into three independent subsets corresponding to different clients. The sample overlap rate and feature overlap rate among participants were strictly controlled at 20% to simulate federated learning scenarios under heterogeneous data conditions. (1) In constructing the sample overlap distribution, we first randomly sampled 20% of the global dataset as overlapping samples, which exist in all clients’ local databases. For the remaining 80% of private samples, to prevent severe label distribution shifts caused by data fragmentation, we strictly performed stratified sampling based on the actual credit default label proportions. Through stratified sampling, the 80% private samples were evenly divided into three independent portions and distributed to three separate clients. (2) For constructing the 20% feature overlap, we extracted 20% of the global features as shared features among the three clients. The remaining 80% of features were randomly partitioned into three independent segments, each assigned as private features for one client. The final data partitioning, which enforces a 20% sample overlap and a 20% feature overlap, results in Client1 having 39 features and 46,666 samples, Client2 having 39 features and 46,667 samples, and Client3 having 38 features and 46,667 samples.

### 4.3. Experimental Results and Analysis

#### 4.3.1. Comparison Experiment

The comparative experiment adopts different federated transfer learning algorithms across the same set of three clients, selecting AUC, Accuracy, Precision, Recall, and F1-Score as classification evaluation metrics. The methods compared include FedKT, XGBoost, M3SDA, FedRep, FedProx, and Local, where ‘Local’ serves as a baseline denoting models trained exclusively on localized data without any federated aggregation.The comparative experimental results across the three clients are presented in Table 2.

The results demonstrate that the proposed FedVI model achieves superior performance across multiple evaluation metrics, with bolded figures indicating the optimal outcomes. In real-world financial credit risk control business scenarios, evaluating the superiority of prediction models must first balance the asymmetric costs of two types of forecasting errors: Type I error (false positive: misclassifying non-default customers as defaulters) only results in lost potential interest income, while Type II error (false negative: failing to detect actual defaulters) directly causes significant credit principal losses. Given this asymmetric risk–cost structure, conventional accuracy metrics—which simply calculate overall prediction correctness rates—cannot distinguish between the severity differences of misclassification and missed detection. Recall, which measures a model’s ability to identify all genuine default cases, serves as the core metric for assessing prevention against Type II errors. However, credit datasets inherently suffer from severe class imbalance (i.e., majority non-default samples vastly outnumber minority default cases), causing traditional models to fall into the “majority class trap” by predicting nearly all borrowers as non-defaulters to achieve superficially high accuracy. This leads to catastrophic Type II errors (reflected in low recall rates). Although some comparative models (e.g., XGBoost, M3SDA, and FedKT) achieved near-0.79 accuracy across three client datasets, their recall values remained around 0.50, indicating they would miss nearly half of high-risk defaulters in practical applications—a critical flaw unacceptable in financial risk mitigation. Compared to other federated learning approaches, FedVI proves more effective in credit default prediction. Under federated settings where client data distributions differ substantially, traditional federated optimization algorithms exhibit pronounced adaptability issues. For instance, the classical FedProx algorithm experienced severe performance degradation on Client1 and Client2 (achieving only 0.248 F1-Score on Client2), highlighting the limitations of simple proximal regularization in addressing deep feature space heterogeneity. The FedVI framework maintains remarkable stability across all clients through its meta-learning mechanism and feature graph alignment. Even on Client1 and Client2, where the performance of baseline models fluctuates significantly, FedVI sustained AUC scores of 0.702 and 0.715 on Client1 and Client2 respectively, showcasing robust cross-domain transfer and personalized adaptation capabilities. Furthermore, compared to advanced federated transfer learning methods (e.g., M3SDA and FedRep), FedVI demonstrates competitive advantages. Although M3SDA achieved a slightly higher AUC on Client1, this minor edge fails to translate into meaningful decision-making benefits due to its lower F1-Score (0.472 vs. FedVI’s 0.569). By converting heterogeneous tabular data into standardized image structures and applying targeted local fine-tuning, FedVI bridges the gap between global knowledge representation and local decision boundaries, achieving superior comprehensive classification performance in credit default prediction.

#### 4.3.2. Ablation Experiment

To study the impact of each component of the FedVI model on classification performance, this research conducted ablation experiments by selectively removing certain parts of the model. Specifically, we established an initial baseline model (“Default”) that generated image block positions using random sorting without semantic coloring and employed the basic FedAvg strategy for parameter aggregation. Building on this baseline, we designed three “single-module retention” variants: the “w.imager” variant enabled only with the semantic coloring module, the “w.Meta” variant enabled only with the federated meta-learning module, and the “w.IGTD” variant enabled only with the feature-ranking-based image generation capability. To validate the contributions of individual components, corresponding “single-module removal” experiments were also conducted. Among these, “w.o.IGTD” removed the feature-ranking functionality in IGTD, reverting it to random sorting, to assess whether the model could achieve performance gains by identifying ordered feature space layouts; “w.o.imager” removed the semantic coloring module based on tabular Transformer, forcing the downstream model to rely solely on grayscale images for learning, thereby verifying the informational value of semantic colors; while “w.o.Meta” eliminated the federated meta-learning mechanism, replacing it with conventional FedAvg aggregation, to quantify the impact of advanced parameter aggregation methods on collaborative training efficacy. It should be noted that this study does not set up independent ablation components for “Dual-Stream Schema Alignment” and “Presence Mask Channel,” as these two elements constitute inseparable structural prerequisites of the framework rather than plug-and-play optimization modules. In extreme heterogeneous federated scenarios, without federated dual-stream schema alignment reconstructing a unified global feature space, the heterogeneous feature dimensions across clients cannot be mapped to a consistent two-dimensional tensor with uniform dimensions, leading to physical-level forward propagation failure; similarly, the Mask channel, serving as the structured fourth dimension defining whether features are genuinely present, if forcibly removed, would require altering the input channel count and network architecture definitions of the underlying CNN, thereby violating the strict control principle of “keeping all variables constant except the one being tested” in ablation experiments. The experimental results are shown in Table 3.

Ablation experiments comprehensively analyzed the contributions of each component in the FedVI model to its overall classification performance. The experimental results demonstrate that the complete FedVI model achieves the highest performance metrics when all components are integrated. Specifically, the complete FedVI model attains AUC values of 0.702, 0.715, and 0.719 on Client1, Client2, and Client3, respectively, with F1-Scores of 0.569, 0.624, and 0.624. These findings strongly validate the synergistic effects among the comprehensive components of the complete model. While individual modules can improve certain metrics, only through their integration does the model achieve high generalization and precise decision boundaries when processing heterogeneous data. Through module-wise ablation, we can clearly quantify the specific contribution of each module. The spatial topology contributes significantly (w.o.IGTD): removing IGTD feature ranking in Client1 and Client2 degrades the global feature map into random arrangement, causing catastrophic drops in F1-Score (from 0.569 to 0.380 in Client1 and 0.624 to 0.270 in Client2). This indicates that without ordered two-dimensional spatial arrangement, the model struggles to learn effective local feature interactions. Secondly, semantic mapping provides indispensable implicit financial logic (w.o.imager). Removing the semantic coloring component forces the downstream CNN model to rely solely on single-channel numerical grayscale images for learning, triggering sharp performance declines in F1-Score (from 0.569 to 0.391 in Client1 and 0.624 to 0.273 in Client2). This suggests that lacking semantic coloring still hinders effective feature interaction learning, leading the model to predominantly predict negative samples and causing Recall imbalance and F1-Score decline. Finally, the meta-optimization mechanism is critical for overcoming distribution shift and negative transfer (w.o.Meta). Removing federated meta-learning and adopting conventional federated averaging aggregation results in severe performance degradation across all three clients, with F1-Score dropping to around 0.32. This further confirms that under extreme heterogeneous environments with low sample overlap, traditional parameter averaging fails to effectively perform cross-domain knowledge transfer. In contrast, meta-learning-based initialization anchors can significantly alleviate local model overfitting and effectively prevent negative transfer phenomena. By comparing the results of Client1, Client2, and Client3 horizontally, it is evident that due to the default model’s AUC reaching 0.708, Client3 represents a relatively easy learning task, whereas Client1 and Client2 tasks are more challenging. The proposed FedVI method demonstrates particular advantages in these two difficult tasks (Client1 and Client2), achieving a 14.0% improvement in AUC and an 11.1% increase in F1-Score over the Default baseline for Client1, and a 16.5% improvement in AUC and a 17.2% increase in F1-Score for Client2, attributed to the synergistic effects of its three core components.

#### 4.3.3. Sensitivity Analysis

This study further constructs multiple heterogeneous federated scenarios with varying overlap rates based on existing experiments with 20% sample overlap and 20% feature overlap for sensitivity analysis. The study sets up more extreme scenarios such as no overlap, low overlap, medium overlap, and high overlap, while maintaining consistent client counts, data partitioning strategies, and evaluation metrics. It compares the performance changes of FedVI against methods like FedKT, XGBoost, M3SDA, FedRep, FedProx, and Local. The experiment aims to verify whether FedVI is effective only under fixed 20% overlap rates or can maintain stable performance across broader heterogeneity levels. The sensitivity analysis results on the Lending Club dataset under 0%, 40%, 60%, and 80% overlap rates are reported in Table 4, Table 5, Table 6, and Table 7, respectively.

From the sensitivity analysis results of the Lending Club dataset, FedVI consistently demonstrates stable comprehensive performance across different overlap rates. Under the most extreme 0% overlap setting, FedVI achieves a high average AUC while maintaining strong Recall and F1-Score on most clients, indicating its capability for cross-domain knowledge transfer without relying on extensive public samples or features. As the overlap rate increases to 40% and 60%, FedVI’s F1-Score further improves, suggesting that moderate public information integration enhances the stability of global visual feature maps and client-side knowledge sharing. At 80% overlap, some traditional methods benefit from increased public features and samples, leading to improved AUC; however, FedVI retains competitive advantages in Recall and F1-Score, highlighting that its strengths stem not only from overlapping information but also from its visual semantic tensor and federated meta-learning mechanisms’ adaptability to heterogeneous distributions.The corresponding sensitivity analysis results on the Home Credit dataset under 0%, 20%, 40%, 60%, and 80% overlap rates are reported in Table 8, Table 9, Table 10, Table 11, and Table 12, respectively.

To further validate the generalization ability of FedVI under different data distributions, this paper conducts the same sensitivity analysis on the Home Credit dataset. The results show that as the overlap rate increases from 0% to 80%, FedVI’s average AUC generally rises, achieving more stable classification performance under high overlap conditions. Meanwhile, in low overlap settings such as 0% and 20%, FedVI still maintains a higher F1-Score compared to most traditional federated learning and local training methods. This indicates that FedVI is not only applicable to the Lending Club dataset but also demonstrates strong cross-domain adaptability under new financial credit data distributions. Notably, under scenarios with limited sample and feature sharing, FedVI mitigates performance degradation issues common in traditional methods through its dual-stream Schema alignment, federated IGTD visual canvas, and Reptile-based meta-learning mechanism.

Comprehensive sensitivity analysis across both datasets reveals that FedVI exhibits robust performance across low, medium, and high overlap scenarios. Unlike conventional FedProx methods that rely on simple parameter averaging for cross-client knowledge sharing, FedVI first maps heterogeneous tables into a unified visual semantic space before using federated meta-learning to identify initialization parameters that enable rapid client adaptation. Consequently, even in 0% or low overlap environments, FedVI sustains high Recall and F1-Score; as overlap increases, its overall performance stabilizes further with additional shared information. These results validate FedVI’s applicability across varying heterogeneity intensities and demonstrate its effectiveness extends beyond the 20% overlap experimental setup.

#### 4.3.4. Efficiency Analysis

To evaluate the feasibility of FedVI in resource-constrained federated environments, this paper conducts an efficiency analysis of FedAvg, FedProx, and FedVI. All methods operate under identical configurations: three clients, 4-channel visual semantic tensor inputs, the same number of communication rounds, and equivalent local training epochs. Specifically, all methods use 100 communication rounds, with each client performing 3 local epochs per round and a batch size of 512. Experiments record metrics including average per-round training time, total federated training duration, average evaluation time, final personalized fine-tuning duration, peak GPU memory usage, model size, and estimated communication overhead. To isolate the effect of the federated optimization strategy, FedAvg and FedProx are implemented using the same visual tensor backbone as FedVI. Experimental results are presented in Table 13.

From Table 13, it can be seen that FedVI’s overall training cost remains within an acceptable range. Its average per-round training time is 13.420 s, with a total federated training duration of 1341.952 s, slightly exceeding FedAvg (1150.471 s) but significantly lower than FedProx (1735.524 s). This result indicates that despite introducing a Reptile-style meta-aggregation mechanism, FedVI’s first-order meta-updates did not lead to a substantial increase in training overhead. Compared to FedProx, FedVI reduces computational costs by eliminating repeated calculations of local objective functions’ proximal regularization terms. Regarding communication overhead, all three methods have model sizes of 0.406 MB, with single-round communication data volumes around 2.436 MB, resulting in total communication amounts close to 243.585 MB over 100 rounds. This consistency arises from their shared parameter upload/download communication mode during main training phases—servers broadcast global model parameters, while clients return updated parameters after local training. Notably, despite FedVI’s architecture incorporating visual semantic tensors and meta-learning mechanisms, its main training phase maintains the same communication complexity as FedAvg and FedProx without additional round-level communication burdens. In terms of computational resources, FedVI’s peak GPU memory usage is 0.743 GB, comparable to FedProx’s 0.744 GB and significantly lower than FedAvg’s 0.933 GB. This confirms the effectiveness of its compact CNN backbone network and four-channel small-sized visual tensor design in reducing memory consumption. Performance evaluations show that FedVI achieves optimal results across AUC (0.698), F1-Score (0.578), and Recall (0.581). Compared to FedAvg, FedVI attains higher Recall and F1-Score through approximately 16.6% longer training time; compared to FedProx, it delivers superior classification performance while shortening training cycles. It is worth noting that pre-processing steps such as dual-stream Schema alignment, federated IGTD canvas construction, table Transformer semantic coloring, and four-channel tensor generation in FedVI are offline one-time operations not participating in iterative communication rounds. Thus, the primary additional overhead lies in offline visual representation building, whereas the main federated training process maintains communication complexity equivalent to baseline methods. Overall, FedVI demonstrates improved cross-domain heterogeneous scenario classification performance while maintaining acceptable training time, communication overhead, and computational resource consumption, providing feasibility evidence for its practical application in resource-constrained federated financial modeling scenarios.

#### 4.3.5. Significance Test

To further verify the statistical reliability of the experimental results, this paper conducts a statistical significance test on each model using 20% overlap data from the Home Credit dataset based on five independent runs. Specifically, this study calculates the mean and standard deviation of each model’s performance metrics (AUC, F1-Score, Accuracy, Precision, and Recall) and employs paired t-tests to compare differences between each model and the Local Baseline. The significance level is set at *p* < 0.05. The experimental results are presented in Table 14.

From Table 14, FedVI achieves F1-Score and Recall values of 0.5756 ± 0.0037 and 0.5767 ± 0.0133 respectively, both being the highest among all models, with statistically significant improvements over the Local Baseline (p < 0.01). This indicates that FedVI can consistently enhance the identification capability of default samples across multiple independent runs, rather than being attributable to random initialization or data partition fluctuations. In contrast, although M3SDA attains higher AUC results, its F1-Score and Recall are notably lower, standing at 0.2894 ± 0.0024 and 0.4009 ± 0.0080 respectively, suggesting its ranking performance fails to effectively translate into identifying minority-class default samples. Similarly, FedKT and XGBoost exhibit high Accuracy but recall rates around 0.5000, indicating a tendency to favor majority classmajority-class predictions. For financial default prediction tasks, misclassifying default clients typically incurs higher costs than falsely flagging normal clients; thus, F1-Score and Recall better reflect a model’s practical business value. Overall, statistical significance tests further validate FedVI’s stability and effectiveness under scenarios with low sample overlap and heterogeneous features.

## 5. Discussion

This paper proposes a novel cross-domain personalized modeling paradigm for heterogeneous tabular data, aiming to reduce the reliance on high overlap rates of overlapping features and samples inherent in traditional federated transfer learning. The superior performance of FedVI under extreme data heterogeneity stems from its abandonment of the conventional “pattern matching” and “sample alignment” processes, which are inherently challenging in tabular data analysis. Such rigid alignment often leads to significant loss of valuable private information, distribution shift, or even negative transfer. In contrast, FedVI transforms cross-domain tabular data into a unified “visual feature space” for efficient collaborative modeling and knowledge transfer.

Existing methods like HeteroFL [11] and FedMA [12] are constrained by rigid structural or hierarchical alignment requirements. FedVI employs a dual-stream feature alignment mechanism to reconstruct global feature maps across statistical and semantic dimensions, enabling heterogeneous features to be mapped into visual space without sharing raw data. This design eliminates dependency on homogeneous model architectures. The availability of third-party public datasets as knowledge distillation mediators is a common assumption in FedMD [14] and pFedKT [24], but such conditions are rarely met in real-world scenarios. By extending the IGTD algorithm [20] to federated settings, FedVI leverages Gram matrix aggregation to reconstruct global feature correlations. Clients’ independent samples are projected onto a unified 2D visual canvas while preserving privacy. Low sample overlap rate remains a challenge in heterogeneous federated learning. FedVI discards traditional federated aggregation strategies, instead adopting a Reptile-based federated meta-learning algorithm. Global models find initialization parameters friendly to all clients’ fine-tuning, mitigating overfitting caused by high data heterogeneity—a design aligned with MAML’s [25] multi-task learning principles.

The study has limitations requiring future work. Although sensitivity analysis covers multiple overlap rates, the experiments are still based on controlled synthetic partitions with three clients; future work will examine larger-scale, real multi-institutional deployments. Additionally, current experiments involve three clients; future studies will test scalability in larger federated networks.

## 6. Conclusions

This paper addresses the dual challenges of feature space heterogeneity and low sample overlap rate in cross-domain federated learning for financial institutions, proposing a novel federated transfer learning framework, FedVI, that integrates visual representation of features and meta-learning mechanisms. The framework innovatively maps heterogeneous tabular data into multi-channel images through a tabular-to-image conversion, capturing both structural features and semantic information. It then employs a federated meta-learning module to achieve deep cross-domain feature adaptation and rapid fine-tuning, successfully establishing an end-to-end federated knowledge transfer system. Extensive comparative and ablation experiments validate FedVI’s superior performance in complex heterogeneous scenarios, particularly demonstrating significant improvements in AUC, recall, and F1 scores under highly heterogeneous data conditions, showcasing its robustness and generalization capabilities. Compared to traditional federated transfer learning methods, FedVI effectively mitigates negative transfer caused by cross-domain data heterogeneity, providing valuable technical support and practical guidance for multi-institutional collaborative modeling.

## Figures and Tables

**Table 1:** Dataset description.

Category	Feature	Description
Borrower Information	home_ownership	Home ownership status (Available in C0, C1, C2)
	yearly_income	Annual income (Exclusive to Client1)
	emp_title/emp_length	Job title (C0) vs. Employment length (C2)
	dti	Debt-to-income ratio (Available in C0, C1, C2)
	verification_status	Income verification status (Available in C0, C1, C2)
	zip_code/addr_state	Geographic location info (Exclusive to Client1)
	application_type	Individual or joint application (Exclusive to Client1)
Loan Characteristics	loan_amnt	The listed amount of the loan applied for (All Clients)
	int_rate/interest_rate	Interest rate (C0, C2 use ‘int_rate’; C1 uses ‘interest_rate’)
	installment	Monthly payment amount (Exclusive to Client1)
	term	Number of payments on the loan (Exclusive to Client2)
	grade/sub_grade	LC assigned loan grade (C2) vs. Sub-grade (C0)
	purpose/title	Loan purpose (C0) vs. Loan title (C2)
	issue_d	The month in which the loan was funded (Exclusive to Client1)
	funded_amnt/funded_amnt_inv	Total amount funded (C2) vs. Funded by investors (C0)
	disbursement_method	Method of disbursement (Exclusive to Client2)
Credit History	open_acc/num_sats	Number of open credit lines (All Clients)
	revol_util/bc_util	Revolving utilization (C0) vs. Bankcard utilization (C1)
	total_bc_limit	Total bankcard credit limits (All Clients)
	mort_acc	Number of mortgage accounts (All Clients)
	inq_last_12m /inq_last_6mths	Inquiries in last 12 months (All) vs. 6 months (C1)
	pub_rec/pub_rec_bankruptcies	Public records (C0) vs. Bankruptcies (C0)
	delinq_2yrs/delinq_amnt	Delinquencies in 2 years (C2) vs. Delinquency amount (C0)
	num_tl_90g_dpd_24m	Accounts 90+ days past due in last 24 months (All Clients)

**Table 2:** Comparative Experimental Results.

Model		FedKT	XGBoost	M3SDA	FedRep	FedProx	Local	FedVI
	AUC	0.687	0.618	**0.708**	0.694	0.571	0.706	0.702
	Accuracy	0.792	0.792	**0.794**	0.784	0.354	0.603	0.610
Client1	Precision	0.616	0.644	**0.683**	0.640	0.526	0.597	0.598
	Recall	0.501	0.515	0.513	0.561	0.514	0.629	**0.648**
	F1-Score	0.445	0.480	0.472	0.561	0.301	0.546	**0.569**
	AUC	0.689	0.666	0.711	0.691	0.615	0.703	**0.715**
	Accuracy	0.792	0.792	**0.795**	0.777	0.308	0.607	0.730
Client2	Precision	0.584	0.396	0.677	**0.624**	0.498	0.602	0.617
	Recall	0.502	0.500	0.527	0.565	0.512	0.620	**0.636**
	F1-Score	0.447	0.442	0.502	0.567	0.248	0.536	**0.624**
	AUC	0.695	0.597	0.719	0.706	0.716	0.719	**0.719**
	Accuracy	0.792	0.792	**0.796**	0.780	0.670	0.625	0.705
Client3	Precision	0.622	**0.720**	0.685	0.639	0.608	0.604	0.619
	Recall	0.504	0.502	0.533	0.570	0.643	0.643	**0.659**
	F1-Score	0.450	0.448	0.512	0.574	0.575	0.565	**0.624**

Note: Bold values indicate the best performance for each metric under the corresponding client.

**Table 3:** Ablation Experiment Results.

Model		Default	w.imager	w.Meta	w.IGTD	w.o.IGTD	w.o.imager	w.o.Meta	FedVI
	AUC	0.562	0.550	0.682	0.633	0.686	0.685	0.604	**0.702**
	Accuracy	0.581	0.685	0.366	0.754	0.463	0.413	0.605	0.610
Client1	Precision	0.533	0.544	0.543	0.579	0.604	0.573	0.557	0.598
	Recall	0.530	0.513	0.549	0.548	0.555	0.581	0.565	**0.648**
	F1-Score	0.458	0.433	0.324	0.525	0.380	0.391	0.510	**0.569**
	AUC	0.550	0.577	0.668	0.605	0.653	0.661	0.511	**0.715**
	Accuracy	0.712	0.632	0.367	0.736	0.308	0.310	**0.767**	0.730
Client2	Precision	0.522	0.509	0.544	0.563	0.264	0.577	0.457	**0.617**
	Recall	0.518	0.517	0.549	0.532	0.533	0.547	0.509	**0.636**
	F1-Score	0.452	0.418	0.326	0.482	0.270	0.293	0.459	**0.624**
	AUC	0.708	0.715	0.719	0.712	0.719	0.717	0.712	**0.719**
	Accuracy	0.667	0.666	0.362	0.642	0.505	0.476	0.663	**0.705**
Client3	Precision	0.612	0.615	0.576	0.604	0.603	0.594	0.612	**0.619**
	Recall	0.628	0.630	0.559	0.645	0.605	0.616	0.643	**0.659**
	F1-Score	0.578	0.578	0.328	0.579	0.460	0.461	0.589	**0.624**

Note: Bold values indicate the best performance for each metric under the corresponding client.

**Table 4:** Sensitivity analysis results of the Lending Club dataset under 0% overlap rate.

Model		FedKT	XGBoost	M3SDA	FedRep	FedProx	Local	FedVI
Client1	AUC	0.661	0.690	0.504	0.673	0.476	0.699	**0.707**
	Accuracy	0.792	0.788	0.479	0.781	0.742	0.604	0.534
	Precision	0.433	0.627	0.499	0.618	0.389	0.594	0.593
	Recall	0.500	0.531	0.499	0.540	0.498	0.629	**0.636**
	F1-Score	0.442	0.515	0.442	0.530	0.424	0.547	**0.515**
Client2	AUC	0.662	0.690	0.534	0.675	0.435	0.690	**0.698**
	Accuracy	0.792	0.796	0.445	0.777	0.650	0.600	0.767
	Precision	0.461	0.722	0.512	0.617	0.420	0.588	0.618
	Recall	0.500	0.518	0.518	0.548	0.480	0.627	0.588
	F1-Score	0.442	0.483	0.427	0.544	0.416	0.548	**0.596**
Client3	AUC	0.655	0.672	0.527	0.681	0.670	0.680	**0.700**
	Accuracy	0.792	0.791	0.351	0.779	0.487	0.580	0.788
	Precision	0.502	0.619	0.514	0.615	0.573	0.584	0.546
	Recall	0.500	0.504	0.515	0.543	0.584	0.580	0.503
	F1-Score	0.442	0.453	0.407	0.536	0.437	0.476	0.454

Note: Bold values indicate the best performance for each metric under the corresponding client.

**Table 5:** Sensitivity analysis results of the Lending Club dataset under 40% overlap rate.

Model		FedKT	XGBoost	M3SDA	FedRep	FedProx	Local	FedVI
Client1	AUC	0.703	0.645	0.692	0.713	0.576	0.712	**0.727**
	Accuracy	0.793	0.792	0.572	0.783	0.385	0.585	0.664
	Precision	0.651	0.896	590	0.641	0.533	0.600	0.615
	Recall	0.508	0.500	0.636	0.568	0.530	0.625	**0.668**
	F1-Score	0.461	0.442	0.541	0.571	0.361	0.528	**0.606**
Client2	AUC	0.700	0.697	0.652	0.697	0.587	0.702	**0.709**
	Accuracy	0.794	0.794	0.522	0.777	0.240	0.623	0.703
	Precision	0.634	0.700	0.573	0.626	0.543	0.604	0.610
	Recall	0.511	0.514	0.607	0.566	0.509	0.626	**0.643**
	F1-Score	0.467	0.474	0.501	0.568	0.208	0.555	**0.615**
Client3	AUC	0.704	0.652	0.687	0.708	0.709	0.713	**0.718**
	Accuracy	0.794	0.792	0.559	0.784	0.630	0.596	0.754
	Precision	0.650	0.674	0.590	0.644	0.605	0.600	0.631
	Recall	0.512	0.501	0.635	0.572	0.635	0.633	**0.636**
	F1-Score	0.469	0.444	0.531	0.577	0.566	0.543	**0.633**

Note: Bold values indicate the best performance for each metric under the corresponding client.

**Table 6:** Sensitivity analysis results of the Lending Club dataset under 60% overlap rate.

Model		FedKT	XGBoost	M3SDA	FedRep	FedProx	Local	FedVI
Client1	AUC	0.711	0.715	0.701	0.724	0.671	0.713	**0.731**
	Accuracy	0.795	0.798	0.547	0.783	0.352	0.616	0.690
	Precision	0.675	0.725	0.590	0.651	0.579	0.607	0.609
	Recall	0.518	0.527	0.635	0.597	0.564	0.634	**0.650**
	F1-Score	0.482	0.499	0.524	0.604	0.341	0.554	**0.612**
Client2	AUC	0.710	0.698	0.671	0.724	0.667	0.714	0.722
	Accuracy	0.795	0.796	0.563	0.783	0.648	0.630	0.683
	Precision	0.676	0.697	0.578	0.646	0.585	0.602	0.613
	Recall	0.517	0.525	0.618	0.585	0.599	0.640	**0.659**
	F1-Score	0.479	0.497	0.529	0.590	0.554	0.568	**0.613**
Client3	AUC	0.713	0.692	0.694	0.728	0.710	0.710	0.709
	Accuracy	0.795	0.797	0.549	0.783	0.648	0.651	0.761
	Precision	0.665	0.723	0.588	0.648	0.607	0.606	0.628
	Recall	0.519	0.522	0.631	0.594	0.640	0.638	0.617
	F1-Score	0.484	0.491	0.524	0.601	0.579	0.579	**0.622**

Note: Bold values indicate the best performance for each metric under the corresponding client.

**Table 7:** Sensitivity analysis results of the Lending Club dataset under 80% overlap rate.

Model		FedKT	XGBoost	M3SDA	FedRep	FedProx	Local	FedVI
Client1	AUC	0.716	0.734	0.714	0.775	0.693	0.713	0.714
	Accuracy	0.795	0.799	0.566	0.796	0.506	0.634	0.759
	Precision	0.670	0.732	0.599	0.686	0.590	0.608	0.628
	Recall	0.523	0.530	0.648	0.650	0.610	0.636	0.620
	F1-Score	0.493	0.506	0.540	0.653	0.478	0.567	**0.623**
Client2	AUC	0.718	0.728	0.694	0.776	0.686	0.726	**0.759**
	Accuracy	0.796	0.799	0.547	0.801	0.423	0.641	0.748
	Precision	0.682	0.737	0.587	0.691	0.587	0.616	0.628
	Recall	0.526	0.530	0.630	0.633	0.594	0.647	0.637
	F1-Score	0.499	0.507	0.522	0.641	0.414	0.576	0.632
Client3	AUC	0.718	0.730	0.719	0.776	0.720	0.718	0.724
	Accuracy	0.795	0.800	0.566	0.797	0.650	0.647	0.567
	Precision	0.668	0.750	0.601	0.688	0.612	0.610	0.603
	Recall	0.525	0.530	0.651	0.645	0.645	0.646	**0.653**
	F1-Score	0.495	0.505	0.541	0.650	0.583	0.582	0.542

Note: Bold values indicate the best performance for each metric under the corresponding client.

**Table 8:** Sensitivity analysis results of the Home Credit dataset under 0% overlap rate.

Model		FedKT	XGBoost	M3SDA	FedRep	FedProx	Local	FedVI
Client1	AUC	0.621	0.620	0.465	0.605	0.480	0.626	**0.638**
	Accuracy	0.919	0.919	0.892	0.918	0.917	0.897	0.907
	Precision	0.460	0.460	0.486	0.486	0.469	0.529	**0.570**
	Recall	0.500	0.500	0.495	0.500	0.499	0.513	**0.519**
	F1-Score	0.479	0.479	0.486	0.480	0.480	0.497	**0.520**
Client2	AUC	0.658	0.688	0.578	0.666	0.487	0.685	**0.699**
	Accuracy	0.919	0.919	0.919	0.917	0.913	0.918	0.841
	Precision	0.460	0.460	0.460	0.527	0.470	0.589	0.570
	Recall	0.500	0.500	0.500	0.502	0.499	0.504	**0.607**
	F1-Score	0.479	0.479	0.479	0.485	0.480	0.488	**0.581**
Client3	AUC	0.658	0.673	0.431	0.667	0.672	0.683	**0.690**
	Accuracy	0.919	0.919	0.919	0.916	0.869	0.892	0.907
	Precision	0.460	0.460	0.460	0.599	0.582	0.595	**0.610**
	Recall	0.500	0.500	0.500	0.509	0.561	0.549	0.541
	F1-Score	0.479	0.479	0.479	0.499	0.550	0.549	**0.553**

Note: Bold values indicate the best performance for each metric under the corresponding client.

**Table 9:** Sensitivity analysis results of the Home Credit dataset under 20% overlap rate.

Model		FedKT	XGBoost	M3SDA	FedRep	FedProx	Local	FedVI
Client1	AUC	0.678	0.586	0.652	0.654	0.531	0.673	**0.678**
	Accuracy	0.919	0.919	0.846	0.916	0.916	0.824	0.880
	Precision	0.460	0.960	0.550	0.609	0.477	0.565	0.578
	Recall	0.500	0.500	0.566	0.509	0.500	0.569	**0.569**
	F1-Score	0.479	0.479	0.556	0.499	0.481	0.547	**0.573**
Client2	AUC	0.703	0.589	0.669	0.693	0.556	0.718	**0.725**
	Accuracy	0.919	0.919	0.847	0.916	0.911	0.825	0.894
	Precision	0.460	0.602	0.557	0.616	0.500	0.579	0.604
	Recall	0.500	0.500	0.578	0.509	0.502	0.610	0.574
	F1-Score	0.479	0.480	0.565	0.498	0.486	0.571	**0.585**
Client3	AUC	0.676	0.662	0.657	0.657	0.660	0.672	**0.685**
	Accuracy	0.919	0.919	0.852	0.914	0.848	0.685	0.883
	Precision	0.460	0.585	0.550	0.582	0.573	0.545	0.585
	Recall	0.500	0.501	0.562	0.511	0.567	0.612	0.573
	F1-Score	0.479	0.480	0.558	0.502	0.548	0.505	**0.578**

Note: Bold values indicate the best performance for each metric under the corresponding client.

**Table 10:** Sensitivity analysis results of the Home Credit dataset under 40% overlap rate.

Model		FedKT	XGBoost	M3SDA	FedRep	FedProx	Local	FedVI
Client1	AUC	0.685	0.623	0.687	0.680	0.566	0.686	**0.688**
	Accuracy	0.919	0.919	0.865	0.916	0.916	0.851	0.910
	Precision	0.460	0.460	0.573	0.614	0.485	0.586	0.607
	Recall	0.500	0.500	0.584	0.510	0.500	0.564	0.529
	F1-Score	0.479	0.479	0.578	0.501	0.481	0.538	0.536
Client2	AUC	0.708	0.639	0.709	0.710	0.534	0.724	**0.733**
	Accuracy	0.919	0.919	0.856	0.915	0.845	0.856	0.907
	Precision	0.585	0.460	0.575	0.620	0.482	0.596	**0.621**
	Recall	0.500	0.500	0.601	0.515	0.500	0.592	0.550
	F1-Score	0.479	0.479	0.585	0.509	0.478	0.565	**0.565**
Client3	AUC	0.689	0.684	0.709	0.676	0.692	0.695	**0.700**
	Accuracy	0.919	0.919	0.862	0.915	0.850	0.825	0.911
	Precision	0.460	0.960	0.576	0.613	0.585	0.582	0.618
	Recall	0.500	0.500	0.524	0.514	0.582	0.587	0.534
	F1-Score	0.479	0.479	0.543	0.507	0.560	0.550	0.552

Note: Bold values indicate the best performance for each metric under the corresponding client.

**Table 11:** Sensitivity analysis results of the Home Credit dataset under 60% overlap rate.

Model		FedKT	XGBoost	M3SDA	FedRep	FedProx	Local	FedVI
Client1	AUC	0.701	0.692	0.669	0.738	0.565	0.672	**0.696**
	Accuracy	0.919	0.919	0.858	0.913	0.841	0.877	0.826
	Precision	0.460	0.817	0.559	0.657	0.532	0.582	0.565
	Recall	0.500	0.501	0.571	0.542	0.510	0.547	**0.612**
	F1-Score	0.479	0.481	0.564	0.550	0.500	0.540	**0.576**
Client2	AUC	0.715	0.704	0.704	0.748	0.549	0.709	0.737
	Accuracy	0.919	0.919	0.837	0.913	0.826	0.900	0.842
	Precision	0.650	0.710	0.567	0.652	0.511	0.621	0.583
	Recall	0.500	0.500	0.605	0.543	0.500	0.544	**0.634**
	F1-Score	0.479	0.480	0.577	0.551	0.483	0.546	**0.598**
Client3	AUC	0.699	0.693	0.660	0.732	0.678	0.684	0.701
	Accuracy	0.919	0.919	0.861	0.913	0.805	0.905	0.861
	Precision	0.460	0.710	0.560	0.653	0.563	0.597	0.574
	Recall	0.500	0.500	0.569	0.545	0.598	0.524	0.590
	F1-Score	0.479	0.480	0.564	0.553	0.552	0.519	**0.580**

Note: Bold values indicate the best performance for each metric under the corresponding client.

**Table 12:** Sensitivity analysis results of the Home Credit dataset under 80% overlap rate.

Model		FedKT	XGBoost	M3SDA	FedRep	FedProx	Local	FedVI
Client1	AUC	0.723	0.717	0.716	0.786	0.642	0.715	0.731
	Accuracy	0.919	0.919	0.780	0.918	0.825	0.910	0.851
	Precision	0.711	0.778	0.562	0.698	0.563	0.647	0.585
	Recall	0.500	0.501	0.641	0.574	0.548	0.529	0.625
	F1-Score	0.479	0.481	0.565	0.592	0.525	0.528	**0.598**
Client2	AUC	0.724	0.718	0.717	0.792	0.634	0.727	0.743
	Accuracy	0.919	0.919	0.800	0.918	0.838	0.916	0.897
	Precision	0.615	0.848	0.566	0.703	0.554	0.660	0.618
	Recall	0.500	0.501	0.637	0.576	0.528	0.513	0.584
	F1-Score	0.479	0.481	0.573	0.594	0.513	0.503	**0.597**
Client3	AUC	0.726	0.728	0.722	0.786	0.725	0.721	0.739
	Accuracy	0.919	0.919	0.805	0.914	0.836	0.875	0.869
	Precision	0.714	0.960	0.566	0.689	0.588	0.601	0.598
	Recall	0.500	0.501	0.634	0.583	0.625	0.586	0.621
	F1-Score	0.479	0.480	0.576	0.598	0.584	0.580	**0.607**

Note: Bold values indicate the best performance for each metric under the corresponding client.

**Table 13:** Calculation efficiency comparison table.

Metric	FedAvg	FedProx	FedVI
Experimental setting			
Rounds	100	100	100
Local Epoch	3	3	3
Batch	512	512	512
Efficiency (lower is better)			
Train/Round (s)	**11.505**	17.355	13.420
Total Train (s)	**1150.471**	1735.524	1341.952
Eval/Round (s)	**3.804**	4.319	4.424
Fine-tune (s)	**166.654**	193.313	195.401
Resource & communication			
GPU (GB)	0.933	0.744	**0.743**
Size (MB)	0.406	0.406	0.406
Comm. (MB)	243.585	243.585	243.585
Performance			
AUC	0.694	0.695	**0.698**
F1	0.564	0.576	**0.578**
Recall	0.562	0.575	**0.581**

Note: Bold values indicate the best result for each metric. For training time, evaluation time, fine-tuning time, GPU memory, model size, and communication cost, lower values are better; for AUC, F1-Score, and Recall, higher values are better.

**Table 14:** Statistical significance test.

Model	AUC	F1-Score	Accuracy	Precision	Recall
M3SDA	**0.7580 ± 0.0026** ***	0.2894 ± 0.0024***	0.8405 ± 0.0032 ***	0.2265 ± 0.0034 ***	0.4009 ± 0.0080 ***
FedRep	0.6562 ± 0.0022 ***	0.5100 ± 0.0010 n.s.	0.9118 ± 0.0006 n.s.	0.5767 ± 0.0046 n.s.	0.5138 ± 0.0006 n.s.
FedKT	0.7004 ± 0.0003 **	0.4790 ± 0.0000 *	**0.9193 ± 0.0000** n.s.	0.5263 ± 0.0913 *	0.5000 ± 0.0000 *
XGBoost	0.6283 ± 0.0224 **	0.4790 ± 0.0001 *	**0.9193 ± 0.0000** n.s.	0.4730 ± 0.0298 **	0.5000 ± 0.0000 *
FedVI	0.6969 ± 0.0013 *	**0.5756 ± 0.0037** **	0.8772 ± 0.0162 **	0.5838 ± 0.0101 n.s.	**0.5767 ± 0.0133** **
FedProx	0.6125 ± 0.0134 ***	0.5046 ± 0.0123 n.s.	0.9105 ± 0.0058 n.s.	0.5043 ± 0.0060 **	0.5180 ± 0.0099 n.s.
Local	0.6984 ± 0.0007	0.5193 ± 0.0241	0.9106 ± 0.0105	**0.6649 ± 0.0753**	0.5253 ± 0.0182

Note: Bold values indicate the best mean performance for each metric. * *p* < 0.05, ** *p* < 0.01, *** *p* < 0.001; n.s. = not significant. Values are reported as Mean ± Std over 5 independent runs. Stars indicate paired t-test significance against Local_Baseline. FedVI is the proposed method.

## Data Availability

The data presented in this study are available in Kaggle at All Lending Club loan data and Home Credit Default Risk.
